# Supplementary material for: Are carbon emissions trading and green financial instruments synergistic? -Comprehensive quantitative research based on content analysis
Source: PLoS One. 2024 Mar 7;19(3):e0298601. doi: 10.1371/journal.pone.0298601 (PMC10919601; doi:10.1371/journal.pone.0298601)
Supplement: S1 Data — (ZIP) [file pone.0298601.s001.zip › Data/地级市Z_F工作报告.zip!/░▓╗╒/│n╓▌╩╨/└┤╫╘╣1⁄2╓┌║┼í╛┬φ┐╦╩2╛▌═°í┐_2008.docx]

各位代表：

现在，我代表市人民政府，向大会作政府工作报告，请予审议，并请市政协委员和其他列席人员提出意见。

一、过去五年发展回顾

过去的五年，在市委的正确领导下，在市人大、市政协的监督、支持下，市政府团结带领全市人民，以邓小平理论和“三个代表”重要思想为指导，全面贯彻落实科学发展观，抢抓机遇，乘势而上，全力推进经济和社会又好又快发展，取得了新成绩。

预计2007年全市生产总值443.9亿元，比上年增长12.8%；财政收入45.5亿元，增长30.3%；全社会固定资产投资236.8亿元，增长58.4%；社会消费品零售总额126亿元，增长16.8%；外贸进出口总额4.75亿美元，增长20%；城镇居民人均可支配收入10900元，增长14.4%；农民人均纯收入3821元，增长18.2%；金融机构存款余额376亿元，增长15.7%；金融机构贷款余额259亿元，增长11.1%。

经过五年艰苦奋斗，全市呈现出发展质量提高、发展速度加快、发展后劲增强、人民生活改善的良好局面，发展成效好于上个五年。2003年至2007年，全市生产总值年均增长9.2%，比上个五年（下同）高4.4个百分点；财政收入年均增长21.7%，高20个百分点；全社会固定资产投资年均增长32.3%，高21.4个百分点；社会消费品零售总额年均增长11.3%，高5.6个百分点；外贸进出口总额年均增长34.5%，高22个百分点；城镇居民人均可支配收入年均增长11.9%，高6.4个百分点；农民人均纯收入年均增长11.4%，高9.7个百分点；金融机构存款余额年均增长19.6%，高6.6个百分点；金融机构贷款余额年均增长9.7%，高6.5个百分点。在经济发展的同时，教育、文化、卫生、计划生育等各项社会事业取得了长足进步。

（一）明确发展目标，确立发展思路。思路决定出路。经过调查研究，市政府坚持以人为本，以改善民生、促进社会和谐稳定为根本目标，制定了全市经济社会发展总体思路：以科学发展观统揽全局，以改革开放为动力，坚定不移地实施东向发展、工业强市和城镇化三大战略。即：以东向发展战略为指引，大力推进全民创业，突出招商引资，突出做大做强工业，并带动一三产业协调发展；以构建滁来全大滁城、开发一批工业园区和培植一批经济强镇为龙头，提升城镇化水平，推动县域经济崛起，促进市县共同发展；以农民增收为核心，改善生产生活条件，推进新农村建设，加快农业农村进步，统筹城乡发展；以财力增强为后盾，加大城乡社会事业、基础设施和社会保障投入，促进全面可持续发展。五年来，全市上下明确目标不动摇，坚定思路不松劲，有效促进了经济社会又好又快发展。

（二）突出招商引资，坚持工业优先。有投入才有产出。五年累计全社会固定资产投资652亿元，是上个五年的2.8倍。把招商引资作为增加投入的主要手段，一二三产全面开放，招商引资先后突破20亿元、40亿元、70亿元、100亿元和200亿元（均不含房地产），累计442亿元，是上个五年的10倍，年均递增78.9%。其中，来自东部发达地区的投资占70%左右；工业投资占70%左右，农林、交通、教育、卫生、旅游、环保、水利等占30%左右。2007年全市规模以上工业企业656户，比2002年增加306户，其中绝大多数为外来投资企业，年产值超亿元企业达90户，增加70户，超10亿元8户、超20亿元2户。五年全市共依法批准使用土地6.8万亩，是上个五年的6.2倍，其中工业项目用地占60%以上，促进了工业强市战略的实施。

（三）拓展滁州中心城市，加速城市化进程。完成滁城总体规划修编。市经济技术开发区、城东工业园五年开发10平方公里，吸纳投资67.5亿元，实现税收6.6亿元，分别是上个五年5.1倍、8.4倍和9.3倍。东扩南移与旧城改造相结合，五年投资58.4亿元：城东、城南新区建设拉开框架，城市环路和新区外延道路形成网络，并与全椒、来安和南谯工业园相衔接，为滁来全组团发展、构建大滁城创造了必要的承载条件；兴建商品房202万平方米，是上个五年的3.2倍；建设和改造50条城市道路，绿化、美化、亮化和各种管线配套到位，并改造小街巷128条；改造了南湖公园、火车站及站前广场；建成市疾控中心、中西医结合医院、职业技术学院、一职高、警察训练基地、检察科技大楼、消防指挥中心和消防三中队、自来水厂沉淀池、垃圾处理厂；建成了大剧院、青少年和妇女儿童活动中心、林业科技大楼、气象科技大楼、清流监狱主体工程；市一院综合楼、城市防洪工程、污水处理厂开工建设；启动了艺校、歌舞团、武警支队搬迁和城南中学新建工程；内城河和北湖水环境综合治理即将开工。五年内，滁城面积扩大15平方公里，占建成区面积的三分之一，发展空间快速拓展，生活条件和投资环境显著改善。

（四）培植经济强镇和工业园区，推动县域经济发展。2005年市委、市政府制定了《关于大力培植经济财政强镇加快县域经济发展的意见》，抓督查促落实，给压力促竞争。列入重点扶持的35个乡镇，财政收入增幅近三年分别达到55.6%、40.2%和41.2%。2006年经省政府批准，各县（市）和南谯区设立了工业园，结束了县域没有工业园的历史，规划面积121平方公里，目前实施开发32.6平方公里，吸纳投资186.7亿元，成为县域经济特别是工业经济的重要增长点。县域城镇建设明显加快。完成了县城和重点镇总体规划新一轮修编。实施中，坚持旧城改造、新区建设、园区拓展三位一体同步推进，拉开了发展框架。五年城镇建成区面积扩大30平方公里，是历史上城镇规模扩张最快的时期。

（五）以农民增收为核心，大力推进新农村建设。全面落实惠农政策，取消农业税，累计发放粮食、良种和农机等补贴近20亿元，严格执行粮价保护政策，调动农民生产积极性。粮食五年四丰收，年产量三创历史新高，2007年产量首破80亿斤大关，达到83.9亿斤。省市级龙头企业发展到58个，农民专业合作组织发展到270个，名牌农产品增加到35个，无公害农产品和绿色食品监控面积扩大到216万亩。植树���林100万亩。农机综合作业水平上升到75%。全面制定了镇（乡）村体系规划，并以此为基础成功推进了乡镇村区划调整，乡镇（办事处）由180个调整到109个，行政村由2193个调整到1438个，为又好又快建设新农村打下了良好基础。以百村十镇试点为示范，推进村庄环境整治，改水40万户，改厕37万户，发展户用沼气2.7万户。改扩建乡镇卫生院79所。农村广播电视村村通工程完成74%。农村道路投资6.23亿元，建成通村水泥路3114公里，其中市财政累计补助1亿多元。投资3.3亿元，完成农村电网二期改造，实现了城乡电网同网同价。水利建设投资11.2亿元，除险加固大中型水库7座、小型水库298座、河湖堤防350公里。实施阳光工程，培训农民8万人。2007年，全市农村全部免除了义务教育阶段学杂费，全面推行了新型合作医疗、最低生活保障等制度。过去的五年，农民群众增收减负，生产条件持续改善，生活质量不断提高，是得到实惠最多的五年。

（六）大力发展第三产业，促进经济协调发展。编制实施城市商业网点规划。引进苏宁、商之都等现代商贸企业，发展新型连锁超市、商品配送中心和电子商务，促进流通业调整业态、完善功能。以实施“万村千乡”市场工程为重点，组建商品配送中心22个、农家店2417个，构建更具活力的农村商品流通体系。编制实施旅游业发展规划，积极创建优秀旅游城市；争取国债资金，整修了琅琊山、全椒神山等重点景区；招商引资开发了来安白鹭岛、天长龙隐山庄等休闲度假区，三星级以上旅游宾馆发展到11家，在建5家；举办欧阳修千年诞辰纪念活动和菊花螃蟹节、凤阳花鼓文化旅游节，加大旅游推介力度。全市旅游总收入由2002年的6.5亿元增加到20亿元。充分发挥金融对经济发展的支撑作用。深化农村信用社改革，转换经营机制，增强服务功能。徽商银行、银联商务、太平保险等金融机构先后入滁发展。五年金融机构贷款余额净增96亿元，利润总额净增23.3亿元，实现扭亏为盈。2007年保险机构保费收入10.6亿元，五年年均增长21.8%。房地产业、交通运输业和中介服务业都有了长足的发展。

（七）深化改革，创新体制机制。五年来，全市上下攻坚克难，完成工业、商业、物资、供销、粮食、医药等400户国有集体企业产权制度改革，多渠道筹集改革资金35亿元，安置职工7.5万人，消化各类债务20亿元，盘活明光酒厂等一大批企业资产64.6亿元。天大集团石油管材股份上市成功。完善政策措施，推动全民创业，私营企业发展到9800户，五年增加5400户；个体私营企业从业人员35.3万人，增加8.3万人，非公有制经济已成为经济发展的重要力量。积极开展农村税费改革和农村综合改革试点，探索农村基层工作新机制。行政机构、人事制度、工资制度、公共财政体制、退役士兵安置等改革稳步推进。

（八）加强基础设施建设，改善城乡发展条件。五年来，交通建设完成投资41.3亿元，比上个五年增长一倍多，等级公路通车里程比2002年末增加618公里，其中高速公路通车里程达270公里，居全省第一。宁洛、宁淮高速公路滁州段建成通车，宁西铁路滁州段进入试运行阶段。电力建设完成投资21.3亿元，形成变电站环网、互联互备的输变电网络，网架规模进入安徽第一方阵。经过几代人的努力，投资23亿元的琅琊山抽水蓄能电站建成并并网发电。通信工程完成投资12.5亿元，固定电话、移动电话和互联网用户分别发展到109.4万户、113万户和9.6万户，行政村全部通电话、通宽带，形成了覆盖全市城乡的通信网络。

（九）全面发展社会事业，大力加强社会建设。积极推进科技成果转化，引导企业开展产学研合作。五年共获得省市级科技成果407项。省级高新技术企业发展到29户。市高新技术创业服务中心建成并投入使用，入孵企业20户。滁州成为全省首个制造业信息化重点城市和电子信息产业基地。投资1200万元，建立了面积达500亩的市农科所新科研试验基地。优先发展教育，城乡义务教育阶段学生学杂费1.45亿元全部免除；五年改造中小学危房73.6万平方米；教育“两基”成果得到巩固；高中阶段毛入学率提升到61%；滁州学院升为本科院校，滁州职业技术学院升为大专院校；38所大中专院校在校生8.7万人，是2002年的3.2倍，职普比例达到4:6。医疗卫生事业发展加快。2003年战胜“非典”，之后加强了公共卫生体系建设，市县两级疾控中心和传染病院（区）项目顺利完成。人口和计划生育工作水平有了较大提升，受到省政府的表彰奖励。2007年人口自然增长率降至5.35‰。文化事业和文化产业加快发展，新闻出版、文艺创作、文化遗产保护取得新成绩。广播电视人口覆盖率提升了40个百分点。广泛开展群众性文体活动。组团参加省十一届运动会，金牌总数超过历届。建成了国家级手球后备人才训练基地。启动了新一轮地方志修编。社区建设得到加强。全市城市社区发展到242个，基本做到有人办事、有钱办事、有地方办事、有章理事。琅琊区被评为全省社区建设示范区。农村社区建设试点进展良好。重视老龄事业发展，健全工作机构，落实优待政策，发展老年教育。积极推进残疾人和妇女儿童权益保障工作。重视国防后备力量建设。荣获全国双拥模范城“三连冠”。人防工程面积五年扩大12倍。民族宗教、外事侨务、档案等其他各项社会事业都有了新的发展。

全力维护社会稳定。认真宣传和贯彻新的信访条例，建立健全矛盾纠纷排查调处机制，持之以恒地开展领导干部开门接访、带案下访活动，信访形势明显好转。突发公共事件预防和应急处理机制初步建立，保障公共安全和处置突发公共事件能力进一步提高。滁州市被省政府授予2005年、2006年安全生产目标管理先进单位。认真开展产品质量和食品安全专项整治，农产品和食品药品生产、流通、消费等环节八大整治任务、十四项百分百要求基本完成。大力推进社会治安综合治理，深入开展“平安滁州”创建活动，努力改善政法部门工作条件，做好司法调解工作，坚决打击违法犯罪活动，有效地维护了社会稳定。

（十）大力推进就业再就业，努力提高社会保障水平。2007年，全市投入5.3亿元，认真落实市委、市政府《关于解决当前群众切身利益若干问题促进和谐滁州建设的意见》，认真实施十二项民生工程，大力推动社会保障制度建设。五年来，通过发展经济、政策扶持、培训服务、提供公益岗位、建立再就业创业园（街）等多种措施，新增城镇就业岗位14.5万个，7.1万下岗人员实现再就业。城镇职工基本养老保险扩面征缴，参保人数扩大到19.8万人。城镇未参加养老保险集体企业退休人员基本生活得到保障。农村五保户供养标准提高，集中供养率提升到20.1%。农村计划生育家庭奖扶政策全面落实。城镇职工失业保险参保人数扩大到22.6万人。城镇职工和居民基本医疗保险参保人数分别扩大到30万人和33.8万人。在全省率先实现新型农村合作医疗制度全覆盖，参合人数302万人，参合率90.4%。建立了城乡特困群众医疗救助和重大传染病人医疗生活救助制度。城镇职工工伤保险和生育保险参保人数分别达到12.5万人和9万人。全市7.3万城镇居民和9.2万农民享受最低生活保障，保障标准不断提高。失地农民基本生活保障和城市流浪乞讨人员生活救助等制度先后建立和不断完善。历年拖欠农民工工资问题基本解决，并有效防止了新的拖欠发生。首批发放大中型水库移民扶助资金5980万元。建立了市县乡三级法律援助体系。全市城镇居民经济适用住房和廉租住房政策已出台实施。2007年市政府提供经济适用住房期房1000套计6万平方米，廉租住房年补贴120万元以上。先后为700户残疾人家庭解决危房或无房问题。面对频发的自然灾害，积极开展生产救灾，五年下拨救灾款1.82亿元，救助灾民140万人次，保证了灾民有饭吃、有衣穿、有房住、有干净水喝、有病能医。红十字会五年募集款物626万元，对扶危济困起到了重要作用。积极开展“两节”送温暖活动，五年发放款物6890万元。

（十一）下大气力整治污染，改善生态环境。编制实施滁州生态建设规划。实行严格的环保准入，大力推行建设项目环境影响评价和“三同时”制度，执行率分别达93%和89%，先后否决了20多亿元高耗能、高污染项目。加强水资源尤其是饮用水源保护。搬迁了影响滁城饮用水安全的市奶牛厂，实施了沙河、清流河、黄栗树等水库、河流黄砂禁采。市县污水处理厂建设全面启动，天长、明光污水处理厂建成并投入试运行。18户重点污染企业实行在线监测，12户重点污染企业治理项目和市环境监控中心、医疗废物处理中心开工建设。建成了5座水和空气质量自动监测站。深入开展环保专项行动。先后对258户企业责令停产整顿，关闭25户。市政府关闭了位于滁城的皖东水泥厂。以推广使用清洁能源、减少污染为主要目的，滁城和天长完成了近200座燃煤小锅炉整治。关闭琅琊山风景区19户采石企业。整顿规范矿产资源开发秩序取得良好成效。

（十二）坚持依法行政，建设服务型政府。以开展保持共产党员先进性教育活动为契机，深入学习邓小平理论和“三个代表”重要思想，牢固树立和落实科学发展观和正确的政绩观。自觉地把政府工作置于市委的领导之下，置于市人大、市政协和人民群众的监督之下。五年来，市政府共办理人大议案10件、人大代表建议329件、政协提案1149件。认真贯彻《行政许可法》等法律法规，依法清理行政许可、审批和收费，重新确认行政许可实施主体，审批项目减少46%。办理行政复议123件。市县两级设立了城市管理行政执法局，相对集中行政处罚权，依法管理城镇。三年来全市共查处违法建设91.8万平方米。针对政府工作中的突出问题，举一反三，依法研究制定指导工作的规范性文件80多件。加强服务型政府建设，推进政务公开，市县两级行政服务中心和政府门户网站全部建成，并向乡镇村延伸，开始形成覆盖市县乡村的四级便民服务网络，投资环境明显改善。2007年，滁州被评为浙商（中国）最佳投资城市和浙商（安徽）最佳服务城市。认真落实政府系统党风廉政建设责任制，强化监察、财政、审计监督，坚决查办违法违纪案件，深入开展治理商业贿赂工作，廉政建设取得积极成效。

各位代表，经过五年艰苦不懈的努力，本届政府五年任期的目标任务已圆满完成，经济社会发展趋好趋快，民生显著改善。主要有以下几个特点：一是投入力度大，经济增长明显加快。全市固定资产投资是历史上最多的五年，保证了经济发展不断提速，生产总值近三年分别增长9.6%、12.1%、12.5%，财政收入、社会消费品零售总额、外贸进出口、城乡居民收入等主要经济指标总量及增速，大幅度超额完成政府五年任期目标。二是经济结构得到优化调整。一二三产业全面增长，其中工业增长更快，五年工业增加值年均增长12.4%，高于上个五年7.9个百分点，高于同期生产总值增幅3.2个百分点。三次产业增加值之比由2002年的29.1:36.6:34.3调整到25.3:40.2:34.5，工业强市战略取得实效。三是人民生活水平明显���高。2007年城镇居民人均可支配收入、农民人均纯收入分别比2002年增长75.7%和71.5%，在衣食住行等生活开支不断提高的同时，城乡居民人均储蓄存款增长1.1倍，社会保障更加健全，五年共发放各类社会保障资金33.7亿元，比上个五年增长2.1倍。四是财政实力明显增强。在免征农业税的情况下，财政收入五年翻了1.4番，增收28.5亿元，五年连跨20亿元、30亿元、40亿元三个台阶，提前两年完成任期目标。财政收入占生产总值的比重由2002年的7%上升到10.4%。五是各项社会事业全面协调发展。全市财政用于各项事业发展的支出五年累计143.3亿元，比上个五年增加77.3亿元。其中：教育支出增长1.1倍，卫生支出增长1.3倍，支农（含水利）支出增长1倍，科技支出增长0.6倍，文化、体育、广播电视支出增长0.8倍，政法支出增长0.9倍。六是市、县两级共同发展、整体快速攀升。财政是经济的综合反映。经济发展反映到财政上，2007年，市级财政收入18.7亿元，比2002年增加12.8亿元，增长2.1倍；县级财政收入26.8亿元，增加15.7亿元，增长1.4倍。市县两级经济财政均结束了较长时期低增长的状况。

各位代表，过去的五年，是全市干部群众理清思路、改革创新的五年，是风正气顺、同心同德的五年，是奋力爬坡、突破徘徊的五年，是改善民生、构建和谐的五年，是成效显著、信心倍增的五年。在此，我谨代表市人民政府向全市442万人民，向人大代表、政协委员，向离退休老同志，向各民主党派、工商联、无党派人士和各人民团体，向驻滁解放军指战员、武警官兵，向国家和省驻滁单位，向所有参与、支持和关心滁州发展的海内外朋友，表示衷心的感谢和崇高的敬意！

五年来，我市经济社会发展和政府工作还存在许多困难和问题。主要表现在：经济总量不大，工业化和城镇化水平不高，人均水平低，预计2007年全市人均生产总值9903元，人均财政收入1030元，分别比全省平均水平低1590元和532元；就业和社会保障压力增大，农民增收的长效机制尚未完全形成，相当多城乡群众生活困难；城乡基础设施仍较薄弱，群众生产生活条件亟待改善；各级财政仍然较为困难，县乡财政对上级转移支付的依赖程度很高，公共服务的保障能力不强；环境保护、节能减排、安全生产还存在许多薄弱环节；政府依法行政水平亟待提高，群众对机关效能建设还有许多不满意的地方。这些困难和问题，是前进中的困难和问题，我们有责任、有决心、更有信心加以解决。

二、未来五年发展目标

党的十七大对2020年全面建成小康社会提出了新的更高要求。政府今后工作的指导思想是：在市委的坚强领导下，高举中国特色社会主义伟大旗帜，认真贯彻党的十七大精神，坚持以科学发展观统揽全局，围绕以人为本、改善民生、促进民享的根本目的，继续坚定不移地实施东向发展、工业强市和城镇化三大战略，更加注重社会建设，更加注重各项事业全面发展，更加注重城乡统筹发展，奋力实现全市人民学有所教、劳有所得、病有所医、老有所养、住有所居、文化进步、社会和谐的发展目标。

目前滁州经济社会发展低于全省平均水平，到2020年要实现与全省同步建成小康社会的目标，滁州经济社会发展必须好中求快，快于全省平均水平，加速崛起步伐。为此，市政府提出未来五年全市经济社会发展的预期目标是：生产总值年均增长13.5%以上，三次产业之比调整到13:52:35，财政收入年均增长24.5%，全社会固定资产投资年均增长16%，社会消费品零售总额年均增长14%，外贸进出口总额年均增长20%，城镇居民人均可支配收入年均增长10.5%，农民人均纯收入年均增长8.2%；人口自然增长率控制在6.5‰以内，万元生产总值能耗明显下降，城乡生态环境明显改善，社会全面进步。

（一）经济发展好中求快，夯实民生基础

科学发展观的核心是以人为本。我们要坚持经济发展好中求快、又好又快，为人民群众日益增长的物质文化需求奠定物质基础。按照未来五年经济发展主要预期目标，到2012年，全市生产总值要达到940亿元，比2007年增长1.2倍，年均增幅比前五年高4.3个百分点。

一要狠抓有效投入，支撑经济发展好中求快。加大投入仍然是滁州发展的首要途径。全市五年固定资产投入达到1800亿元，比前五年增加1150亿元。在各级政府投资、争取上级投资和鼓励民间投资、利用资本市场直接融资的同时，把招商引资作为增加投入的主要途径，五年招商引资1500亿元。

二要突出工业强市，狠抓经济结构的战略性调整。通过招商引资、发展非公有制经济等途径，五年工业项目到位资金达到1000亿元以上，规模以上工业企业增加到1200户，其中亿元以上200户，10亿元以上40户，20亿元以上20户，50亿元以上5户，支持定远盐化工项目建成投产，争取大唐电力项目批准建设。着力打造家电、机械、纺织、化工、农副产品深加工、非金属矿深加工等六个百亿元产业。到2012年，全市工业增加值占生产总值的比重达到45%以上，比2007年提高10个百分点。

三要着力打造经济发展平台。大力发展市经济技术开发区和县级工业园区，新辟琅琊工业集中区，五年工业园区面积扩大50平方公里，入园项目投资强度和科技含量明显提高。继续培植经济强镇，加快县域经济崛起。到2012年，35个重点乡镇财政收入均超5000万元，比2007年增加31个，其中超8000万元20个，增加17个，超亿元10个，增加7个。

四要提升城镇化水平。以滁城为中心，以来安、全椒两个县城为两翼，构建经济意义上的大滁城。结合乡镇区划调整，加强县城和重点集镇规划建设，培植经济强镇，形成各具特色的经济强镇群，促进人口向城镇集中。做大做强房地产业和第三产业，拉动消费，培植新的经济支柱。到2012年，建筑业增加值达到66亿元，比2007年增长1.9倍；第三产业增加值达到329亿元，增长1.2倍。五年内全市城镇人口发展到210万人，城镇化水平上升到47%。

五要加快城乡基础设施建设。交通建设完成投资78亿元。积极支持建设马鞍山至扬州等6条高速公路滁州段工程，高速公路通车里程突破400公里。完成滁州至南京快速通道建设和104国道、311省道、101省道滁州段路面提升改造。形成更为完备的供电、供水、供气、通信、商业、旅游等基础设施网络。

六要深化改革，创新体制机制。全面完成国有集体企业改制任务，加快推进事业单位改革，大兴全民创业，大力发展非公有制经济，增强内在发展活力。各县（市、区）全部建立中小企业信用担保机构，缓解中小企业融资难问题。

七要加大科技投入，鼓励自主创新。加快数字滁州建设。建立以企业为主体、市场为导向、产学研相结合的技术创新体系。支持企业技术改造、使用新技术和创建新品牌，提升综合竞争力。

八要继续推进社会主义新农村建设。加快发展现代农业和农村二三产业，推进农业产业化经营，提升市场化水平，构建农民增收的长效机制。城市基础设施向农村延伸，政府公共服务向农村覆盖。到2010年，建设3000公里通村水泥路，全面完成村村通工程。新建800个小型客运站（亭），实现路通车通。开工建设滁河防洪工程。完成28座中型水库和312座小型水库除险加固。完成滁城和全椒、天长城市防洪工程建设。解决111万农村人口饮水安全问题。各县（市）自身财力用于水利投入每年均不低于800万元，两区均不低于500万元。

（二）狠抓就业再就业，促进劳有所得

就业是劳动者自立自强自尊的基础，是劳动者的第一需要，是民生之本。因此，经济发展首要的目的是创造更多的就业和增收机会。认真贯彻《就业促进法》，确保未来五年全市新增城镇就业15万人以上，城镇登记失业率控制在4%以内。市县两级都要至少建立1所规范的人力资源市场。加快推进失业登记、技能培训鉴定、就业指导、职业介绍、劳动保障代理等服务。继续实施阳光工程，五年培训农民10万人以上。建成4个高技能人才培训基地，9个再就业和农村劳动力培训基地，创建1所技师学院、1—2所高级技工学校。全面推行职业资格证书制度。到2012年末，高技能人才在技术性从业人员中所占比例达到10%。落实就业和再就业的各项扶持政策，重点抓好零就业家庭、“4050”人员的就业再就业，更多地为他们提供公益性岗位援助和小额贷款、税费减免、社保补贴、岗位补贴等。积极发展社会福利企业，促进有能力的残疾人就业。强制实施最低工资制度，逐步提高最低工资标准。多渠道筹措失业保险基金，增强支付和调剂余缺的能力，到2012年末全市失业保险参保人数达到26万人。深入贯彻《劳动合同法》，狠抓劳动合同管理，加大劳动执法力度，健全职工工资支付保障机制和增长机制，建立劳动争议调解仲裁机制。

（三）完善保障体系，做到贫有所济、老有所养

要加大养老保险扩面征缴力度，将城镇职工基本养老保险覆盖各类企业职工、个体工商户和灵活就业人员，确保到2012年参保人数突破22万人，基金征缴率保持在90%以上。完善城镇职工基本养老保险市级统筹，逐步做实个人账户，逐步提高养老保障标准。鼓励支持有条件的企业建立年金制度。积极探索建立机关、事业单位和农村居民基本养老保险制度。继续实施农村计划生育家庭奖励扶助制度。完善失地农民社会保障制度，逐步提高保障标准。农村“五保”对象实现应保尽保，生活水平不低于当地群众平均生活水平，到2012年每个乡镇至少建设1所中心敬老院，集中供养率提高到45%以上。完善城乡居民最低生活保障制度，扩大保障范围，提高保障标准。加强灾害应急救援体系建设，努力做到指挥系统健全完善，社会动员及时有效，物资供应方便快捷，灾民生活妥善安排。完善孤老孤残儿童和流浪未成年人救助保护制度，完善救助机构功能。加强老龄工作，保障老年人合法权益。建立健全慈善事业救助管理体制和社会捐赠网络。

（四）加快发展卫生事业，实现病有所医

健康是小康社会的重要内容，关系千家万户的幸福。要着力缓解看病难看病贵问题，不断提高城乡居民的健康水平。

加强公共卫生建设。全面提高疾病预防控制能力，切实做好重点传染病和地方病防治工作。健全突发公共卫生事件应急机制。大力发展妇幼卫生事业。坚持公共医疗卫生的公益性质，强化政府的责任和投入，以公立医疗机构为主导，多种所有制医疗机构共同发展。完善新型农村合作医疗制度，提高参保标准，增加农民受益程度，加大对特困群众医疗救助力度，努力使农民参合比例提升到95%以上。切实抓好乡镇卫生院和村卫生室的标准化改造，确保到2010年末每个乡镇至少有1所标准化卫生院，每个村至少有1所标准化卫生室，全市基本实现乡镇卫生院和村卫生室房屋、设备、技术及管理“四配套”。不断完善城镇职工、居民基本医疗保险制度，不断提高医疗保险标准。全面实施工伤保险，建立市级工伤保险储备金制度。逐步扩大生育保险的覆盖范围。到2012年末，全市城镇职工基本医疗保险、工伤保险和生育保险参保人数分别增加到35万人、19万人和13万人。大力发展城市社区卫生服务，不断完善社区卫生服务机构功能，努力构建城市医院与社区卫生服务机构分工合理、双向转诊的新型医疗服务体系。到2012年，居民到城市社区卫生服务机构首诊就诊率达到50%以上，社区卫生服务人口覆盖率琅琊、南谯两区达到100%，各县（市）城达到80%以上，基本实现小病在社区、大病进医院的目标。到2012年，全市每千人拥有病床上升到2张以上，每千人拥有卫生技术人员上升到2.7人以上，城乡新招录合格医护人员3000名，重点向农村倾斜，配齐配强医疗卫生队伍。对乡镇医护人员制定住房政策，使人才留得住，更好地为农民服务。

（五）优先发展教育事业，实现学有所教

教育关系滁州人民的未来，必须坚持把教育放在优先发展的战略位置。巩固和发展基础教育。到2012年，城乡幼儿入园率达到70%以上，适龄儿童入学率达到99.5%以上，初中入学率达到98%以上，高中阶段毛入学率超过70%。办好来安、全椒特教学校，残疾少年儿童随班就读、完成义务教育。大力发展职业教育，力争五年中市及每个县、市、区新组建1—2所职业学校，全市高中阶段教育职普比例达到1:1。提升高等教育的办学规模和水平。完成滁州职业技术学院的搬迁扩建和滁州学院新校区建设，继续做好凤阳师范升大专工作，支持安徽科技学院做大做强，支持滁州电大发展现代远程教育。搬迁扩建市委党校。积极发展成人教育，构建全民终身学习的学习型社会。

完善教育投入保障机制。坚持教育公益性质，以政府投入为主，依法保证财政性教育经费增长，保证学生人均经费明显增长。政府继续承担城乡义务教育阶段学生学杂费，对农村学生免费提供教科书，建立健全助学金、奖学金和资助特困生制度。全面完成农村中小学危房改造。优化学校布局，均衡配置公共教育资源。大力推进教育现代化建设，力争到2012年城镇学校教育设备达到国家标准，农村中学教育设备基本满足教学需要，乡镇中心小学建有计算机教室，村小学配备成套教学光盘和播放设备，开通城乡各类学校网络视频系统。鼓励、支持和引导社会力量投资办学。到2012年，省市示范高中发展到35所，市级以上重点职业学校发展到15所，各类示范特色学校发展到100所。全面提高教育教学质量。坚持育人为本、德育为先，德智体美全面发展，减轻中小学生课业负担。加强师德建设和业务培训。五年中，全市按编制新招录合格教师2740名，重点向农村倾斜，配齐配强教师。对乡镇教职工制定住房政策，使人才留得住，更好地为农民服务。

（六）增加各类住房供应量，促进住有所居

安居才能乐业。各级政府要一直把解决住房问题摆在重要位置。到2012年，全市城镇人均住房面积达到30平方米，比2007年增加5平方米。加大房源供应，五年兴建1000万平方米商品房，比前五年增加1倍以上。同时，市、县两级全面实施经济适用住房和廉租住房制度，逐步改善城镇低收入家庭住房困难。五年完成150万平方米经济适用住房、30万平方米廉租住房建设。努力创造条件，逐步缓解城市中等收入家庭住房困难。鼓励企业特别是工业企业兴建职工公寓，为在岗职工提供基本的住宿条件。要制定政策措施，对商品房开发限期开工、限期竣工交付，制止圈地屯地、拖延开发、屯房涨价行为。要正确引导住房消费，倡导量力而行、理性适度的消费观念。要完善城镇公共交通，使居住区分布均匀，不断降低房屋地段差价。通过以上综合措施，努力改变住房价格完全靠市场调节的局面，使住房消费能力不同的群众各有所选、各得其所。要有计划地实施沿淮等低洼易涝地区群众房屋迁建。要结合集镇经济发展，通过宅基地置换和户籍制度改革等办法，鼓励农民居住向集镇集中。要加大力度，尽力解决城乡残疾人、农村低收入家庭无房、危房问题。

（七）加强先进文化建设，丰富群众精神生活

充分发挥先进文化的教育感化作用。围绕构建以“八荣八耻”为主要内容的社会主义核心价值体系，深化文化体制改革，积极发展文化事业和文化产业，繁荣新闻出版、广播电视、文学艺术和哲学社会科学事业，坚持正确导向，弘扬先进价值观、人生观、世界观和社会正气。广泛开展文化、科技、卫生“三下乡”等活动。加强对地方文化的挖掘和保护，重视文物和非物质文化遗产保护工作。加强文化市场管理，坚持不懈地开展“扫黄打非”，严禁网吧等娱乐性经营场所接纳未成年人。加强文化基础设施建设。市县两级积极创造条件，力争到2012年不同程度地建设一批档案馆、图书馆、体育场（馆）、影剧院、青少年和妇女儿童活动中心、工人文化宫、展览（博物）馆、村文化室、社区和乡镇综合文化站等文化体育设施。大力改善市艺校、歌舞团、黄梅剧团、京剧团发展条件。广泛开展群众性文化艺术和体育活动，开展文学艺术创作评比，倡导健康向上的情趣爱好，丰富精神生活。

（八）建设资源节约型、环境友好型社会，促进生态文明

正确处理经济社会发展与人口、资源、环境的关系，全面推进生态市建设。稳定低生育水平，着力解决出生人口性别比失衡等突出问题。不断提高计生专干生活待遇。努力建设环境友好型社会。加强森林资源保护，植树造林，森林覆盖率提高到27%。严格执行环境影响评价和“三同时”制度，从源头上治理污染。加快对淮河、滁河、清流河等流域污染治理，改善水环境。高度重视自然生态和饮用水源保护，控制农村面源污染。到2012年，滁城和各县城污水处理厂和垃圾无害化处理厂全部建成运营。积极探索循环经济发展模式，按照减量化、再利用、资源化的原则，加强资源综合利用。搞好土地节约集约利用，保护耕地。整顿和规范矿产资源开发秩序。推进天然气等清洁能源使用和城市集中供热，倡导节约能源资源的消费方式，努力建设节约型社会。

（九）完善社会管理，维护社会安定和谐

要坚持以人为本，创新社会管理理念和管理方式，最大限度地激发社会创造力，最大限度地增加和谐因素，最大限度地减少不和谐因素。加强民主法制建设，全面推进依法治市。加强社区建设，充实人员，提高待遇，改善社区服务条件。开展和谐社区、和谐村镇、和谐企业、和谐家庭创建活动，构筑促进和谐的社会基础。加强国防教育、国防动员，进一步做好双拥共建工作。妥善处理人民内部矛盾，完善信访制度，健全社会纠纷调处机制，促进人际关系的和谐。完善突发事件应急管理机制，建立健全社会预警体系，提高保障公共安全和处置各类突发事件的能力。全面落实安全生产、社会治安综合治理和维护稳定的各项措施，努力形成社会安定、环境安全、百姓安宁的良好局面。

各位代表，未来的五年，是为全面建设小康社会继续打基础的五年，是推动跨越式发展、大力改善民生的五年。市政府要紧紧围绕以人为本、改善民生、促进民享这个核心，坚持发展为了人民、发展依靠人民、发展普惠人民，全方位、创造性地开展工作，真正体现权为民所用、情为民所系、利为民所谋。

三、2008年发展任务

2008年是全面贯彻十七大精神的第一年，是改革开放三十周年，也是新一届政府开局之年。做好今年工作，对未来发展意义重大。中央今年实施稳健的财政政策和从紧的货币政策，加强和改善宏观调控。这对我们来说既是挑战，更有机遇。我们要准确把握，积极适应国家宏观调控要求，坚持好字优先、好中求快，实现经济社会又好又快、跨越式发展，进一步改善民生，促进社会和谐稳定，为五年发展开好局、起好步。综合各种因素，2008年全市经济社会发展的主要预期目标是：生产总值增长13.5%以上，财政收入增长24.5%，全社会固定资产投资增长26.2%，社会消费品零售总额增长16%，外贸进出口总额增长20%，城镇居民人均可支配收入增长10.5%，农民人均纯收入增长8.2%，城镇登记失业率控制在4%以内，人口自然增长率控制在6.5‰以内，单位生产总值能耗降低4%，主要污染物排放量减少3.9%。

（一）继续大力实施工业强市战略，突出抓好招商引资

以东向发展战略为指引，全面贯彻落实市委、市政府《关于加快推进工业强市战略的决定》，坚定不移地把工业强市作为经济跨越发展的核心战略。制定和实施工业发展指标考核体系和考评办法，层层设立工业发展专项基金，建立工作推进机制。围绕发展家电、机械、纺织、化工、农副产品深加工、非金属矿深加工等六大支柱产业，编制产业发展规划。积极参与实施省工业振兴计划和“861”行动计划，积极参与建设合芜滁马汽车、家电工业走廊和“两淮一蚌”重化工走廊。认真贯彻市委、市政府《关于进一步深化滁宁合作的意见》，积极参与区域经济分工，搞好基础设施对接，融入南京都市圈。要咬定招商引资不放松，不断掀起招商引资新热潮，确保今年招商引资突破280亿元。坚持一二三产业全面开放，招商引资好中选优。继续把工业特别是先进制造业作为招商引资重点。加快开发区、工业园区和重点乡镇工业集中区建设，力争今年开发面积扩大12平方公里。积极采取土地置换、城乡挂钩、提高投资强度、“腾笼换鸟”等措施，切实解决建设用地不足的瓶颈制约。强化招商引资竣工投产项目考核，促进项目落实和加快建设进度。

通过招商引资、启动民资，鼓励企业技术改造，鼓励企业进入资本市场直接融资，规模扩张与产业升级并举，引进大项目，发展大企业，培育大产业，延伸产业链条，形成产业集群。今年全市规模以上企业要突破750户，其中亿元以上企业突破110户；力争新上投资额500万元以上项目500个，其中5000万元以上项目90个，亿元以上项目30个。

（二）拓展城镇发展空间，加速城镇化进程

大力推进市委、市政府《关于加快实施城镇化战略的决定》的贯彻落实。按照滁州中心城市总体规划要求，完善近期建设规划、控制性详细规划和修建性详细规划。按照建设清流河城市风光带、提升城市品位的要求，完成清流河综合整治规划和两岸开发规划。加快城南新区建设，启动开发区二期工程和琅琊工业集中区建设，推动城市东扩南移西跨北上，拓展城市规模，推进大滁城开发建设。投资8.8亿元，新建城南大道等7条道路，改续建扬子东路等19条道路，基本建成45平方公里城市路网框架。兴建商品住房60万平方米。编制城市公交发展规划，扩大公交覆盖范围。搬迁丰乐油库，拆除城内小铁路并将其改建成城市公路，建设丰乐亭公园。建成城南新区供水工程。加快建设市污水处理厂和垃圾填埋场配套工程建设。开工建设“两湖一河”综合整治工程，加快建设防洪工程西撇洪沟。加强重点道路绿化美化亮化，搞好老城区城中村改造，改善人居环境。

加强县城和小城镇规划与建设。以县域镇村体系规划为基础，结合乡镇区划调整，完成乡镇总体规划和50%行政村村庄规划编制工作。继续把培植经济强镇和工业园区作为推进县域经济发展的重要抓手，强化扶持措施，强化督查考核，推动其快速上台阶，并发挥产业吸纳带动作用，让更多的群众包括农民群众就近就业、就近安居。积极推进农民宅基地使用权的转让、置换，改善基础设施，促进农民居住向城镇和规划居住点集中。

按照“相对集中、重心下移”的原则，不断完善城镇管理体制。健全市县两级行政执法部门职能，增强基层街道、乡镇在城镇管理中的主体和基础作用，鼓励和支持群众参与城镇社区管理，形成“条块结合、以块为主，职责明确、协调配合，统分结合、高效运行”的城市管理体制。以文明城市创建活动为抓手，提高城镇文明程度。

（三）大力发展现代农业，加快社会主义新农村建设

坚持把发展现代农业、繁荣农村经济、增加农民收入作为农村工作的首要任务。不断推进农业结构调整。继续实施国家优质粮产业工程，大力开展水稻产业提升行动和小麦高产攻关，加强大型商品粮基地建设。认真落实生猪和奶牛生产扶持政策，优化水产养殖结构，推动规模养殖和标准化养殖，争取今年养殖业在农业中的比重上升到45%以上。面向长三角地区建设无公害蔬菜等生产基地，同时抓好滁菊等地方特色产品生产。充分利用区位、资源优势，打造粮食、苗木花卉等区域性物流中心。全面实施集体林权制度改革及配套改革，推进林业产业发展。建设20万亩生物柴油原料林基地。着力培育农产品品牌、加工型龙头企业和农民专业合作组织，推进产业化经营。今年省市级龙头企业要发展到70个，农民专业合作组织发展到320个。健全农技推广体系，发展农业信息化服务，实施农机富民工程，提高农业科技应用水平和创新能力。加强农产品质量安全检测检验体系建设。实施阳光工程，培训转移农民3万人，并鼓励务工人员回乡创业。

推广新农村建设“百村十镇”试点示范经验，按照“走平坦路、喝干净水、上卫生厕、住整洁村”的要求，全面整治农村生产生活环境。加快农村饮水安全工程建设，确保今年解决22万农村人口的安全饮水问题。实施农村生态家园富民工程，控制农村面源污染。发展户用沼气7000户。继续加大农业综合开发、江淮分水岭综合治理和农村扶贫开发的力度。切实把水利工程建设摆上农村可持续发展的战略位置抓紧抓好。总结去年防汛抗洪经验，组织人力、物力和财力，确保今年汛前完成水毁工程建设和隐患工程除险加固。抢抓国家加大水利设施投入的机遇，积极编报项目，全力争取投资。今年要完成《滁河干流防洪治理可行性研究报告》的编报工作，抓好驷马山、女山湖两大灌区续建工程建设，争取沿淮洼地治理项目、沿江排涝泵站项目和天长焦涧水库、定远芝麻水库等10座中型水库除险加固工程如期开工建设。

认真落实党的农村各项方针政策。完成农村土地承包换证工作，妥善解决农村土地纠纷，稳定和完善土地承包关系。按照依法自愿有偿的原则，指导农村土地健康有序流转，探索符合本地实际的适度规模经营模式。加强和改进农民负担监督管理和农村财务管理，规范“一事一议”，引导农民发展农村公益事业。

（四）加快发展现代服务业，提升第三产业层次和规模

发展现代服务业是推进工业化、城镇化的内在要求，必须把提升服务业水平作为经济结构调整的重要方向，大力培育与先进制造业相配套的信息咨询、技术交易、金融保险、仓储物流、售后服务等生产服务业，努力构建全方位、多层次的大流通格局。运用新技术新业态改造传统商贸服务业。着力引进知名流通企业来滁发展。市经济技术开发区重点支持宇培工业仓储物流项目建设。优化商业网点布局，大力发展连锁经营和电子商务。深化农村商品流通体制改革，发挥“万村千乡”市场工程作用，完善农村商品流通和消费服务网络。着力调整外贸结构，努力扩大家电、电子元器件、汽车及配件、石油管材、化工、轻纺等优势产品出口。争取国家在滁设立海关和商检机构。继续开展优秀旅游城市创建活动。加快旅游基础设施建设，培育特色品牌和精品线路，强化区���旅游联动，加强推介和宣传，提升旅游接待能力，努力提高旅游经济和社会效益。认真落实国家房地产政策，推动房地产业健康发展。今年全市要兴建商品住房200万平方米。

（五）加快城乡基础设施建设，改善生产生活条件

交通建设完成投资8亿元。着力抓好交通东向对接，开工建设滁宁快速通道、滁州至汊河、滁州至定远道路改造工程。突出滁城中心城交通枢纽地位，启动大西环和滁来全快速通道规划等前期工作。完成蚌宁高速滁州连接线、宁淮高速汊釜连接线建设。积极推进马滁扬高速公路、明徐高速公路、凤淮高速公路、宿扬高速公路建设的前期工作，做好京沪高速铁路滁州段建设的地方配合工作。农村道路“村村通”工程建设今年确保完工1200公里以上，市财政继续每公里补助4万元。电力建设完成投资5.2亿元。扩建500千伏清流变电站，新建220千伏宝桥、110千伏花园、菱溪、汊河、关塘、东阳七个输变电工程，完成220千伏清流至滁州变电站输电线路工程建设，进一步完善供电网络，提高供电能力。发展邮政事业，积极拓展速递物流类业务。实施通信能力提升工程，加强信息化基础设施建设。

（六）大力发展社会事业，促进经济社会协调发展

在加快经济发展的同时，拿出更多的财力支持社会事业发展。优化教育结构。推进素质教育，促进学生德智体美全面发展。完善义务教育经费保障机制，全面实施农村中小学标准化建设工程、现代远程教育工程、薄弱学校建设工程，促进义务教育均衡发展。加快普及高中阶段教育，大力发展职业教育，提高高等教育规模和质量。落实高校和中职学校学生资助政策。加快滁州职业技术学院新校区建设进度，完成城南中学一期工程建设。全市新招录教师600名。积极推进科技创新，争创全省创新型试点城市和国家级电子信息产业园。启动市高新技术创业服务中心二期工程建设。加强重大疾病防控，提高医疗救治能力，开展城乡医疗卫生改革试点。完善新型农村合作医疗制度，落实新的补助标准。完成31个标准化乡镇卫生院、205个标准化村卫生室、27个城市社区卫生服务机构建设。加快建设市第一人民医院综合楼。全市新招录700名医护人员。实施贫困白内障患者复明工程。全面推进农村社区建设，进一步提升城市社区建设水平。加快建立计划生育“村为主”、性别比治理、社会抚养费征收三大工作机制，加强流动人口管理，落实农村计划生育家庭奖励扶助政策，稳定低生育水平。加强文化市场管理，继续开展扫黄打非和网吧专项治理。搞好第三次文物普查及非物质文化遗产普查。滁州大剧院、妇女儿童和青少年活动中心建成并对外开放。完成市歌舞团、市艺校迁建主体工程。启动市黄梅剧团和京剧团迁建工程。实现广播电视信号村村通。依法加强档案管理。完成新的市档案馆建设筹备工作。各县、市根据情况，启动档案馆、图书馆、体育场（馆）等文体设施的规划、选址和立项报批工作。借北京奥运会东风，广泛开展群众性文体活动，发展竞技体育。深入开展“五五”普法，切实加强社会主义荣辱观和社会公德、家庭美德、职业道德教育。加强国防动员，扎实推进民兵预备役工作，大力发展人民防空事业，继续做好双拥共建工作。广泛开展民族团结进步活动，依法管理宗教事务。大幅增加民族乡村发展专项补助资金。加快发展社会福利事业、慈善事业、残疾人事业和老龄事业。支持工会、共青团、妇联、工商联、科协等人民团体的工作。

（七）全面深化改革，着力增强经济社会发展活力

继续采取多种形式筹集改革资金，严格按政策妥善解决职工安置等问题，推进以产权制度为核心的国有集体企业改革。市本级今年要加快水泥厂政策性破产，启动热电厂改制重组，基本完成物资、轻工、商业、粮食、供销、外贸等系统企业改制任务，推进交通、水利、建筑等企业改革。县、市、区要继续加大力度，实施企业改革攻坚。优化全民创业环境，进一步落实促进非公有制经济发展的各项政策措施，推动非公有制企业做强做大，提高非公有制经济的比重。建立健全公共财政体制，提高公共服务能力。全面实施政府非税收入收缴制度改革，逐步将非税收入纳入国库集中收付。切实搞好金融改革试点，推动金融产品创新和服务创新。扶持邮政储蓄银行发展，引进股份制商业银行，培育农村商业银行。扎实推进社会信用体系建设，完善企业征信系统，开展个人征信建设。全面推行工业用地招标拍卖挂牌制度改革。进一步完善生态补偿机制。加快推进事业单位分类改革。不断深化农村综合改革。切实加强村级财务管理规范化建设，努力消化乡镇村债务，防止发生新的不良债务。

（八）强力推进节能减排，切实保护环境

坚决执行国家产业政策，扶优限劣，淘汰落后，突出抓好重点行业、重点企业节能降耗工作。认真执行建设项目节能评估审查制度、环境影响评估制度和核准程序，把好环保准入关。加强工业园区等区域环评工作。做好项目建设的跟踪监督和竣工项目环保验收，提高“三同时”执行率和验收质量。着力推进淮河、滁河、清流河流域水环境治理。加快市县污水处理厂建设，天长、明光污水处理厂要正式投入运行。完成市环境监控中心和医疗废物处理中心建设。各县市要启动垃圾无害化处理厂建设。深入开展环保专项行动，严肃查处环境违法行为。建立健全节能减排工作责任制和问责制，加强考核和监督，管住增量，调整存量，确保完成节能减排任务。完成整顿和规范矿产资源开发秩序年度任务，推进矿产资源整合，提高资源开发利用水平。

（九）提高社会保障水平，促进社会和谐稳定

加大就业和社会保障工作力度。落实就业再就业各项扶持政策，完善就业服务体系，千方百计增加就业岗位，加大对就业困难人员、大中专毕业生和农村转移劳动力的就业帮扶力度。继续实施消除零就业家庭计划，确保双下岗、双失业、单亲等特殊困难家庭至少有1人就业。年内实现城镇新增就业2.4万人以上，下岗失业人员再就业1万人。进一步扩大基本养老保险、医疗保险、失业保险、工伤保险、生育保险和城乡居民最低生活保障的覆盖范围，并逐步提高保障标准。重点推进社会保障向非公经济组织从业人员、灵活就业人员和农民工延伸。完成城镇居民基本医疗保险全覆盖。完善农村五保户供养、特困群众大病救助、灾民救助、孤残儿童和城市流浪乞讨人员救助制度。新改扩建农村敬老院和五保老人之家88个，确保集中供养率提高到26%以上。今年开始，对年满18周岁以上、丧失劳动能力、且无个人收入来源的残疾人给予生活救助。加强自然灾害的监测、预警和防控，抓好救灾救济工作。切实解决城镇低收入家庭的住房困难。今年，全市今年要完成30万平方米经济适用住房、5万平方米廉租住房建设。扩大廉租住房政策享受范围，提高补助标准。解决部分残疾人家庭无房、危房问题。今年，市政府要研究、出台政策，向城市中等收入且住房困难的家庭提供限价或定价商品房。抓好大中型水库移民后期扶持工作。加强物价监管，努力抑制物价过快上涨。完善法律援助体系。强化劳动执法监察，严格执行最低工资标准，健全农民工工资保障机制。加快人力资源市场和基层劳动保障平台建设。坚决落实安全生产责任制，大力整治安全隐患，严防重特大事故发生。继续整顿和规范市场秩序，完善监管体制和机制，加强食品药品生产、流通、消费等各个环节的安全监管，巩固产品质量和食品安全专项整治成果。严厉打击危害人民群众身体健康和生命安全的非法生产经营活动。加强信访工作，完善利益诉求、权益保障、矛盾调处等机制，积极预防和妥善处置各类群体性事件。加强社会治安综合治理，深入开展基层平安创建活动，实施城乡社区警务改革，建立健全社会治安防控体系，依法打击违法犯罪活动，维护社会安定和谐。今年组建职业制消防队伍。建成市特警支队、车辆管理综合服务中心。开工建设市武警支队新营区、市化学消防中队。

（十）坚持依法行政，加强政府自身建设

加快转变政府职能。在继续搞好经济调节和市场监管的同时，加强社会管理和公共服务职能，把精力更多地放到社会发展和管理上来，把财力物力更多地集中到公共服务上来，把政策的着力点更多地放到解决民生上来。加快推进政企分开、政资分开、政事分开、政府与市场中介组织分开，调整优化机构设置，精简和规范各类议事协调机构及其办事机构，理顺部门分工。大力推行行政审批权相对集中制度，减少和规范行政审批，保障行政审批工作依法规范有序开展。

全面推进依法行政。进一步落实依法行政实施纲要，严格依照法定权限和程序行使权力、履行职责，把依法行政作为政府工作的基本准则，努力建设法治政府。加强行政执法部门建设，健全行政执法体制，规范行政执法行为，推进综合执法。加强行政执法监督，落实行政执法责任制和过错追究制，促进严格执法、公正执法、文明执法。依法接受人大及其常委会的法律监督和工作监督，主动接受政协的民主监督，自觉接受社会各界和人民群众的监督，促进政府依法行政。改进工作方法，认真办好人大议案、代表建议和政协提案。

大力推行政务公开。认真贯彻《政府信息公开条例》，按照依法公开、真实准确、注重实效、方便群众、有利监督的原则，扩大政务公开范围，规范公开内容，丰富公开形式。对运用行政权力办理的与人民群众利益相关的各类事项，都要依法、及时地向社会公开，实行阳光操作。积极推进电子政务建设，拓展网上审批和服务，提升在线办事水平。规范招投标和政府采购，建设市招投标服务中心，将各类招投标逐步纳入中心交易，推进相关部门与所属项目招投标的脱钩。强化行政服务中心建设，进一步完善功能，创新机制，提升窗口审批效能，形成市县乡村四级联动、快捷规范的服务通道。

切实加强政风建设。牢固树立正确的政绩观，多做有利于长远发展、有利于打牢基础的工作，胸怀全局，埋头苦干，追求厚积博发。健全重大问题集体决策制度、社会公示和听证制度，增强工作的科学性、系统性、预见性和创造性。坚持艰苦奋斗、廉洁从政，以制度建设为重点，用制度管人管事管钱，严肃查处违法违纪案件。进一步增强政府的执行力，把这次市人代会确立的发展目标，层层分解，以攻坚克难的精神抓落实，以求真务实的作风抓落实，以健全有效的机制抓落实，以队伍素质的提高抓落实，确保落实到位。

各位代表：新的发展蓝图已展现在我们面前，把蓝图变为现实，是历史赋予我们的神圣使命。我们要在中共滁州市委坚强领导下，紧密团结在以胡锦涛同志为总书记的党中央周围，高举中国特色社会主义伟大旗帜，全面落实科学发展观，继续弘扬小岗村大包干精神，解放思想，同心同德，锐意进取，为滁州经济社会又好又快发展、跨越式发展、和谐发展，为创造滁州人民更加美好的新生活而努力奋斗！
